# Mitochondrial Genomes of Six Snakes (*Lycodon*) and Implications for Their Phylogeny

**DOI:** 10.3390/genes16050493

**Published:** 2025-04-26

**Authors:** Fei Zhu, Anqiong Lu, Ke Sun

**Affiliations:** School of Life Sciences, Guizhou Normal University, Guiyang 550025, China; 18485778038@163.com (A.L.); sunkeke@163.com (K.S.)

**Keywords:** *Lycodon*, mitochondrial genome, phylogenetic relationships, comparative analysis

## Abstract

Background: Colubridae, known to be one of the most species-rich snake families, remains relatively understudied in termshe context of complete mitochondrial genome research. This study provide the first systematic characterization of the mitochondrial genomes of six colubrid species: *Lycodon subcinctus*, *Lycodon rosozonatus*, *Lycodon fasciatus*, *Lycodon gongshan*, *Lycodon futsingensis*, and *Lycodon aulicus*. Method: In this study, mitochondrial genomes were sequenced using Sanger sequencing. The raw data were subjected to quality- filtered withing using Fastp and subsequently assembled into complete mitochondrial genomes via SPAdes. Gene annotation was performed by Tblastn, Genewise (for CDS coding sequences), MiTFi (for transfer RNAs), and Rfam (for ribosomal RNAs). Sequence analyses were conducted with various tools, including MEGA, tRNAscan-SE, DnaSP, MISA, and REPuter. Finally, phylogenetic trees were reconstructed based on 13 protein-coding genes from 14 species. Results:The mitogenomes of these six species ranged from 17,143 to 17,298 bp in length and con-sisted of 13 protein-coding genes (PCGs), 22 transfer RNA genes (tRNAs), 2 ribosomal RNA genes (rRNAs), and 2 control regions. The nucleotide composition of the Colu-bridae mitogenomes was comparable with an A + T composition ranging from 52.1% to 58.8% except for the trnS1 and trnC. All the tRNAs could fold into a stable secondary structure. The Pi and Ka/Ks values suggested that atp8 was the fastest-evolving gene, while cox1 was the most conserved gene. Bayesian inference and maximum likelihood phylogenetic analyses yielded consistent results, with the six sequenced species clus-tering together with their congeneric species. These findings will provide valuable references for further research on the phylogeny of Colubridae.

## 1. Introduction

The *Lycodon* genus, which belongs to the Colubrinae family known for its high species diversity within the suborder Serpentes, is one of the most diversified genera of Asian colubrids [1]. Currently, there are 82 known species globally (Reptile Database; http://reptile-database.reptarium.cz, accessed on 22 April 2025) ranging from central Asia and eastern Iran to southern China, the Indo-Australian Archipelago, Japan, and the Philippines [2,3]. Of these 82 species, a small portion of them have been documented in China [4,5,6,7,8]. The primary characteristics of the genus *Lycodon* include smooth or ridged dorsal scales, vertically elongated pupils, an anteriorly curved and arched maxilla, double rows of subcaudal scales, and scales covering the middle section of the body [2,9]. The genus *Dinodon* was established by Dumeril in 1853, with *D. cancellatum* designated as the type species. Key features of this genus encompass vertically elongated elliptical pupils, double rows of subcaudal scales, and elongated, narrow labial scales. Although the morphological characteristics of *Dinodon* closely resemble those of *Lycodon*, phylogenetic analyses using multiple loci have revealed that *Dinodon* is nested within *Lycodon*. Consequently, the entire *Dinodon* group has been reclassified under *Lycodon* [3,10].

Early studies of the species relationships within genus *Lycodon* were based on morphology, but this approach inevitably led to disagreements [11]. A few studies have reconstructed phylogenetic relationships based on multiple genes (Cyt b, nad4, c-mos, Rag1) [3,12]. Recent studies using molecular data have provided some new insights into the phylogenetic relationships among genus *Lycodon* species. At present, the species relationships among genus *Lycodon* were established based on the mitochondrial Cyt b gene [13,14]. There are slight differences in the phylogenetic trees constructed by the two methods. Due to the lack of complete mitochondrial genome data of the genus *Lycodon*, the relationship among them is still not fully examined.

Mitochondria are organelles present in most eukaryotic cells, playing a critical role in energy production [15]. Animal mitochondrial DNA (mtDNA) exhibits maternal inheritance and possesses a double-stranded circular structure. Its structural simplicity, characterized by the absence of introns and the presence of multiple copies, along with the rarity of recombination events, makes it a vital tool for investigating the molecular systematic evolution and development of eukaryotes [16,17,18]. As an informative molecular marker, phylogenetic relationships based on mitochondrial genomes typically demonstrate greater resolution, reliability, and robustness compared to those of other molecular markers [19]. Consequently, in recent years, the rapid advancement of genome sequencing technology has led to an increasing number of scholars analyzing species’ evolutionary relationships by comparing mitochondrial genomes across different species [20,21].

In this study, we reported the complete mitochondrial organizations and characteristics of six species belonging to the family Colubridae (*L. subcinctus*, *L. rosozonatus*, *L. fasciatus*, *L. gongshan*, *L. futsingensis*, and *L. aulicus*). Then, we described and analyzed the basic features of the mitochondrial genome, nucleotide composition, codon usage bias, nucleotide diversity (Pi), ratios of nonsynonymous (Ka)/synonymous (Ks), simple sequence repeats, and dispersed repeats. In addition, based on 13 protein-coding genes, the phylogenetic trees of 14 species (including 12 *Lycodon* snakes and 2 outgroups) were reconstructed by Bayesian inference (BI) and maximum likelihood (ML) methods. We hope that our study can enable better comprehension of *Lycodon* biodiversity and expand genetic resources for future *Lycodon* comparisons.

## 2. Materials and Methods

### 2.1. Specimen Collection

The data pertaining to the specimens collected in the course of this study are presented in the Table 1, and the collected specimens were soaked in anhydrous ethanol.

### 2.2. Extraction, Sequencing, and Annotation of Mitochondrial DNA

Liver tissues from six samples were excised using sterilized anatomical instruments, preserved in absolute ethanol, and sent to Shanghai Biotechnology Bioengineering Co., Ltd. (Shanghai, China) for sequencing. Sanger sequencing was performed using an Applied Biosystems 3730xl Genetic Analyzer (Thermo Fisher Scientific, Waltham, MA, USA), and the original image data files obtained from sequencing were converted into sequencing sequences through base recognition analysis. The raw data were assessed for quality and filtered for adapters and low-quality sequences using Fastp, resulting in the final clean data. Following the acquisition of high-quality sequencing data, the genomes were assembled using SPAdes v3.15.5 [22] (https://github.com/ablab/spades, accessed on 25 April 2024), incorporating multiple K-mer values [23]. The final complete genome sequence was derived by integrating the splicing results from each K-mer value. For gene annotation, coding sequence (CDS) gene boundaries were determined using Tblastn and Genewise [24] along with the relevant reference database; MiTFi [25] was utilized for tRNA sequence annotation, and Cmsearch from the Rfam database was employed to identify coding rRNA. Ultimately, the complete annotation results and the sequences were submitted to the GenBank database.

### 2.3. Sequence Analyses

Nucleotide composition, codon usage, amino acid composition, and relative synonymous codon usage (RSCU) values were calculated by MEGA 7.0 software [26]. Nucleotide bias was calculated according to the following formulas: AT skew [AT]/[A + T] and GC skew [GC]/[G + C] [27]. The secondary structures of the 22 tRNA genes were predicted using tRNAscan-SE 1.21 [28]. DnaSP 5.1 [29] was used to calculate the non-synonymous substitution rate (ka), synonymous substitution rate (ks), the ratio of ka / ks, and nucleotide diversity (pi) for protein-coding genes. Nucleotide diversity (pi) was plotted using the R-ggplot2 package (v3.5.0) [30]. The MISA software (v2.0) [31] was used to find SSRs in the organelle genome, and REPuter (v3.0) [32] annotated organelle genome repeats.

### 2.4. Phylogenetic Analysis

Phylogenetic trees summarize the genetic relationships among species and delineate their direct evolutionary links [33]. We downloaded a total of 8 complete mitochondrial genomes of Colubridae species from the NCBI database and combined them with mitochondrial genomes of 6 additional species from our study, resulting in an initial dataset comprising 14 species.

This dataset was imported into Phylosuite v1.2.3 software [34] for the extraction of 13 protein-coding genes (PCGs). Subsequently, multiple sequence alignments of each PCG were performed using MAFFT v7.464 software [35]. However, since MAFFT v7.464 does not account for the structure of PCGs, it may introduce alignment errors; therefore, it was necessary to optimize the aligned PCG sequences using MACSE [36]. GBlocks v0.91b was then employed to prune the PCGs, removing sites with alignment errors or multiple substitutions to eliminate phylogenetic noise and retain phylogenetic signals [37]. Next, the concatenation function in Phylosuite v1.2.3 was utilized to combine the individual genes of each species into a multigene sequence, which provides more phylogenetic information. Finally, within Phylosuite v1.2.3, the Best Information Criterion (BIC) and the greedy search strategy implemented in PartitionFinder [38] were employed to select the optimal partitioning scheme and evolutionary model. Besides Partitionfinder, ModelFinder [39] also has the same function. For the maximum likelihood (ML) analysis, ModelFinder identified the General Time Reversible (GTR) substitution model with gamma-distributed rate heterogeneity (+G) and a proportion of invariant sites (+I) as the best-fit model based on BIC scores. Based on the results obtained from PartitionFinder and ModelFinder, the phylogenetic relationships among these 14 species were constructed using Bayesian inference (BI) and maximum likelihood (ML) methods, respectively. IQ-tree v2 software [40] was used to construct the maximum likelihood (ML) phylogenetic tree, and bootstrap support (BS) was assessed using 1000 ultrafast bootstrap replicates. The Bayesian inference (BI) phylogenetic tree was constructed using MrBayes v3.2.6 software [41]. The MCMC iterations were set to 10,000,000 generations, with a sampling frequency of 1000. Four MCMC chains were run, and a Burnin Fraction value of 0.25 was applied to discard the first 25% of the samples. The online website TVBOT (https://www.chiplot.online/tvbot.html) (accessed on 10 May 2024) was used to visualize the phylogenetic trees of BI and ML [42].

## 3. Results

### 3.1. The Mitochondrial Genome Structure and Characteristics

**Figure 1:**
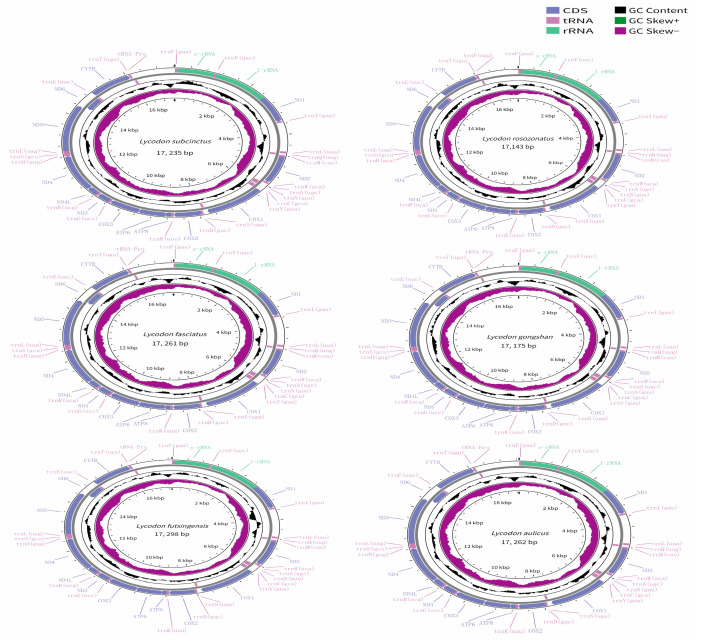
Circular maps of the mitogenomes of *L. subcinctus* (**a**), *L. rosozonatus* (**b**), *L. fasciatus* (**c**), *L. gongshan* (**d**), *L. futsingensis* (**e**), and *L. aulicus* (**f**). Genes are shown in different color blocks. Color blocks being outside the circle indicates that the genes are located on the heavy strand (H-strand); color blocks being within the circle indicates that the genes are located on the light strand (L-strand).

The mitogenomes of the six species of the genus *Lycodon* were typical circular molecules with lengths of 17,235 bp for *L. subcinctus*, 17,143 bp for *L. rosozonatus*, 17,261 bp for *L. fasciatus*, 17,175 bp for *L. gongshan*, 17,298 bp for *L. futsingensis*, and 17,262 bp for *L. aulicus* (Figure 1, Table 2). The six mitogenomes contained 37 genes: 13 protein-coding genes (PCGs), 2 ribosomal RNA genes (12S rRNA and 16S rRNA), 22 transfer RNA genes (tRNAs), and two control regions. The gene arrangement was identical among the six species. Among the 13 PCGs, there were seven NADH dehydrogenase subunits (nad1–nad6), three cytochrome genes (cox1–cox3), two atp synthase genes (atp6 and atp8), and one cytochrome b gene. Among them, the nad6 gene and eight tRNA genes were encoded on the heavy strand (H strand), while the remaining 28 genes were distributed on the light strand (L strand) (Figure 1). As with other snake mitogenomes, they all contained two control regions with lengths ranging from 1011 to 1158 bp. In this region, one was located between trnP and trnF, which involves the replication origin of the H chain and the double-stranded transcription origin; the other was located between trnI and trnL (Figure 1).

There were 6–9 intergenic spacers (IGS) in the complete mitochondrial genomes of the six species, with sizes ranging from 1 to 46 bp in length (Table 3). The longest IGS (46 bp) was identified between the cox1 and trnS2 genes in *L. subcinctus*, but it had the smallest number of IGS (6 bp). Four locations had same IGS across the six mitogenomes: trnL2-trnQ (1 bp-long IGS), trnQ-trnM (1 bp-long IGS) trnY-cox1 (1 bp-long IGS), nad6-trnE (9 bp-long IGS). We also identified five (*L. subcinctus*) to six overlaps distributed in six mitogenomes ranged from 1 to 10 bp (Table 3). The longest overlap region was located between atp8 and atp6 in the six species, which was 10 bp. Four locations had the same overlaps across the six mitogenomes: atp8-atp6 (10 bp-long overlaps), atp6-cox3 (1 bp-long overlaps), trnS1-trnL1 (3 bp-long overlaps) and nad4L-nad4 (1-bp long overlaps). Therefore, the main difference was between nad5-nad6 (5 or 9 bp) and trnF-rrnS (0, 2, or 3 bp).

### 3.2. Nucleotide Composition Analysis

The nucleotide compositions of the six newly sequenced mitogenomes were biased toward A and T, which was similar to that observed in other vertebrates (Table 4). Specifically, the compositions were as follows: *L. subcinctus*: A + T = 58.6%; *L. rosozonatus*: A + T = 59.4%; *L. fasciatus*: A + T = 58.5%; *L. gongshan*: A + T = 58.2%; *L. futsingensis*: A + T = 58.2%; and *L. aulicus*: A + T = 58.2%. According to the A + T skew formula, all six species exhibited inconspicuously positive values varying from 0.173 (*L. subcinctus*) to 0.150 (*L. fasciatus* and *L. aulicus*), while all GC skews were markedly negative, varying from −0.370 (*L. gongshan*) to −0.387 (*L. subcinctus*) (Table 4).

### 3.3. Protein-Coding Gene and Codon Preference Analysis

The entire lengths of the 13 PCGs of the six sequenced mitogenomes ranged from 10,695 to 10,704 bp (Table 2). Each of the PCG sequence exhibited a comparable size, with the nad5 gene being the longest (1770 bp or 1773 bp) and atp8 being the shortest (159 bp). In the six newly sequenced mitogenomes, nad6 was encoded on the L-strand, while other PCGs were on the H-strand (Figure 1). The total A + T content of the PCGs ranged from 58.2% (*L. subcinctus*) to 59.4% (*L. futsingensis*) (Table 5). Based on the comparative analysis, in the 13 PCGs of the six species, all genes except cox1 used the conventional ATN (where N represents T or G) as the start codon ATN (where N represents T or G). In the PCGs of *L. subcinctus*, *L. gongshan*, and *L. aulicus*, the genes atp8, atp6, nad4L, nad5, and nad6 used the termination codon TAA or AGG, while the remaining eight PCGs terminated with an incomplete termination codon (TA and T). In contrast, unlike these three species, the nad4 and nad6 genes of *L. rosozonatus*, *L. fasciatus*, and *L. gongshan* used the TAA and AGA as the termination codons, respectively (Table 2).

**Figure 2:**
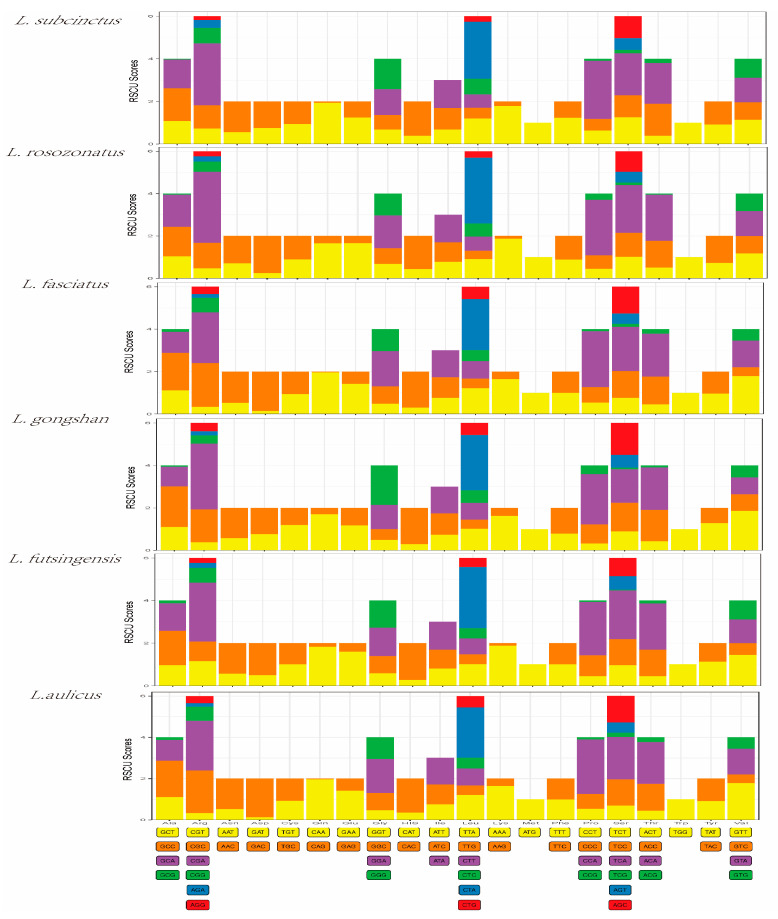
Relative synonymous codon usage (RSCU) values in protein-coding genes (PCGs) of the six *Lycodon* species.

The PCGs in the mitochondrial genomes of *L. subcinctus*, *L. rosozonatus*, *L. fasciatus*, *L. gongshan*, *L. futsingensis*, and *L. aulicus* were composed of 3652, 3643, 3649, 3649, 3648, and 3650 amino acids, respectively. The most frequently used amino acids in the PCGs of these six species was leucine (15.78–15.21%), isoleucine (13.95–15.12%), and threonine (12.67–11.76%), while the least frequently used amino acid was tryptophan (<1%) (Table 6). Codon usage bias analysis refers to the phenomenon of non-random usage of synonymous codons encoding the same amino acid in an organism. It was used to infer the evolutionary relationships between species based on the differences in codon usage preferences. The Relative Synonymous Codon Usage (RSCU) value can measure the frequency of codon usage. As shown in Figure 2, the three codons with the higher RSCU values in the mitochondrial genomes of the six species were CTA, CCA, and CGA, indicating that these three codons were the most frequently used in encoding the mitochondrial genome (Figure 2). The codon usage preferences were similar among the six species.

### 3.4. Nucleotide Diversity (Pi) and Nonsynonymous (Ka)/Synonymous (Ks) Mutation Rate Ratios

**Figure 3:**
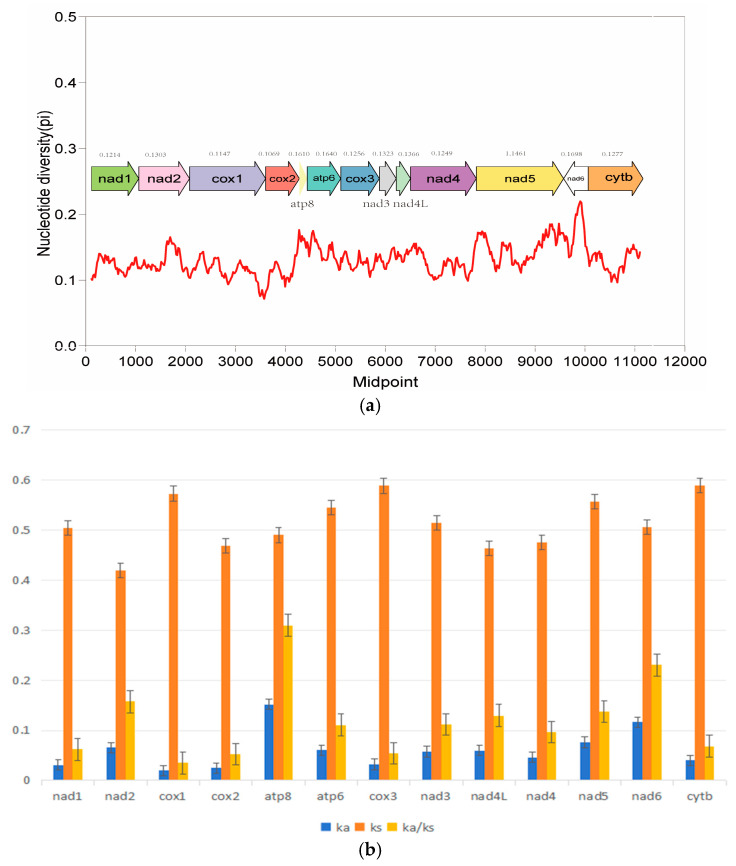
Nucleotide diversity (**a**) and the Ka/Ks ratio (**b**) of PCGs in mitochondrial genomes of 14 species of *Lycodon.* The red line represent the trend of nucleotide diversity variation.

Pi values can be used to evaluate the genetic diversity level of population genomes, reflecting the major variant regions among genomes. In this study, the Pi values of the mitochondrial genomes of the six species were calculated (Figure 3). The results showed that atp8 and atp6 had higher Pi values (>0.16) than other coding genes. On the contrary, cox1 and cox3 showed lower nucleotide diversity values. The Ka/Ks ratio is used to assess the evolutionary rate of 13 PCGs in species (Figure 3). The results showed that the Ka/Ks ratios of all PCGs were less than 1, indicating that these genes were in a state of purification selection. In addition, the Ka/Ks ratio of the atp8 and nad6 gene were much higher than that of other genes and is subjected to the least selection pressure, suggesting it has a higher evolutionary rate. As with the result of nucleotide diversity analysis, the ka/ks value of cox1 and cox3 were smaller than other genes, suggesting that they were relatively conserved among the mitochondrial protein-coding genes due to their slower evolution rate and higher selection pressure.

### 3.5. Analysis of Simple Sequence Repeats and Dispersed Repeats

In this study, simple sequence repeats (SSRs), which consist of 1–6 nucleotide units repeated in tandem multiple times, were analyzed in the six species (Figure S1). The results showed that both *L. subcinctus* and *L. rosozonatus* had 5 SSRs. However, the types of SSRs in *L. subcinctus* were divided into p2 (two nucleotide repeats) and p3 (three nucleotide repeats), while *L. rosozonatus* included p1 (single nucleotide repeat), p2, p3, and p4 (four nucleotide repeats), totaling four types. Additionally, three SSRs were found in each of the other four snake species. Apart from *L. futsingensis*, which had p1, p2, and p3 SSR types, the other three species all had types p2, p3, and p4.

Dispersed repeats are repetitive sequences that are scattered throughout the genome. They include four types: forward (F), reverse (R), complement (C), and palindromic (P). After using REPuter to annotate the repetitive sequences in the mitochondrial genomes, the results showed that the main type of dispersed repeats were F and P, and there were no R or C repeats. *L. rosozonatus* had the highest proportion of F-type repeats, while *L. futsingensis* had the lowest. In contrast, *L. aulicus* had the highest number of P-type repeats, with *L. rosozonatus* having the lowest (Figure S2).

### 3.6. Transfer and Ribosomal RNA Genes

The 22 tRNAs were detected in the six species, with lengths ranging from 57 (trnS) to 73 bp (trnL) and the entire length being between 902 (*L. gongshan*) and 910 bp (*L. rosozonatus* and *L. futsingensis*) (Table 2, Figure 1). Among of 22 tRNAs, eight tRNAs were located on the L-stand, and the other 14 were on the H-stand. The A + T contents of 22 tRNAs spanned from 58.1% (*L. subcinctus*, *L. rosozonatus*, *L. fasciatus*, *L. gongshan*, and *L. aulicus*) to 58.7% (*L. futsingensis*) with a high AT bias and all also exhibited a positive AT skew (0.171–0.185) and negative GC skew (0.138–0.168) (Table 5). The secondary structures of the 22 tRNAs in the six newly sequenced mitogenomes were generated and are presented in Figures S3–S8. All tRNAs could fold into a typical cloverleaf secondary structure, except for trnS1 (L-strand) and trnC (L-strand), which lacked a dihydrouracil (DHU) arm and T-arm, respectively. The trnS1 and trnC of *L. subcinctus*, *L. gongshan*, and *L. futsingensis* could not form a typical cloverleaf secondary structure, while the other three could only be trnS1.

The full size of two rRNAs genes in the six species ranged from 2385 (*L. gongshan*) to 2405 bp (*L. rosozonatus*), and both 12S rRNA and 16S rRNA were encoded on the H-stand. (Table 2, Figure 1) In addition, the nucleotide composition of 12S rRNA and 16S rRNA was calculated separately, and the A + T contents extended from 58.1% (*L. subcinctus*) to 59.2% (*L. futsingensis*) and all showed positive AT skew (0.315–0.340) and negative GC skew (0.208–0.227) (Table 5). The 12S rRNA genes were located between trnF and trnV, with sizes ranging from 926 (*L. subcinctus*, *L. fasciatus*, and *L. aulicus*) to 931 bp (*L. rosozonatus*). The 16S rRNA genes were located between trnV and nad1, with sizes ranging from 1466 (*L. futsingensis*) to 1474 bp (*L. rosozonatus*) (Table 2).

### 3.7. Phylogenetic Analysis

**Figure 4:**
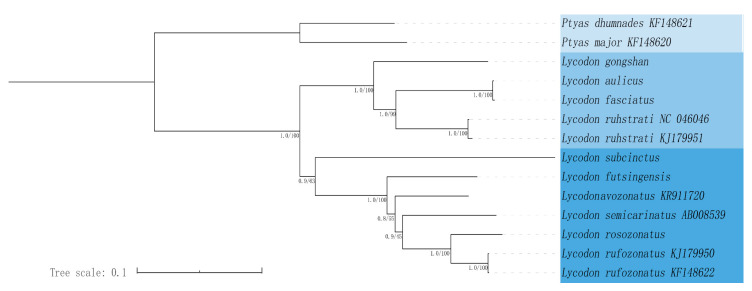
Phylogenetic estimate of relationships within the 14 *Lycodon* species based on the 13 PCGs using Bayesian inference (BI) and maximum likelihood (ML) analyses.

In this study, we conducted a phylogenetic analysis that included 12 species of *Lycodon*, as well as outgroup species from *Ptyas*. Phylogenetic analyses were performed based on the maximum likelihood (ML) and Bayesian inference (BI) methods. The maximum likelihood (ML) and Bayesian inference (BI) trees showed consistent topologies, and most nodes were strongly supported (Figure 4). The result showed that the phylogenetic tree contained two large branches: one consisted of two species of the genus *Ptyas*, and the other consisted of 12 species of the genus *Lycodon*. BI and ML trees showed strong support (BS = 100, PP = 1) for the monophyly of *Lycodon*, and the six complete mitogenomes covering one genera in this study have good clustering in phylogenetic trees. Within the clade including 12 species of *Lycodon*, five species of *Lycodon* (*L. gongshan*, *L. aulicus*, *L. fasciatus*, *L. ruhstrati* NC 046046, and *L. ruhstrati* KJ 179951) formed a strongly supported monophyletic group, in which *L. aulicus* and *L. fasciatus* were closely related, and *L. gongshan* was the sister of the other four species. Another seven species (*L. subcinctus*, *L. futsingensis*, *L. semicarinatus*, *L. flavozonatus*, *L. rosozonatus*, *L. rufozonatus* KJ 179950, and *L. rufozonatus* KF 148622) also formed a single clade, in which *L. subcinctus* was the sister of the other six species, *L. futsingensis* was the sister of the other fice species, and *L. rosozonatus* was the sister of *L. rufozonatus* KJ 179950 and *L. rufozonatus* KF 148622.

## 4. Discussion

In this study, we sequenced and comparatively analyzed the complete mitogenomes of six species and found that the gene order, contents, and base composition were identical to those found in typical vertebrates [16,43]. The length of the mitogenomes of the six species ranged from 17,143 (*L. rosozonatus*) to 17,298 bp (*L. futsingensis*) (Figure 1). The typical metazoan mitochondrial genome size is considered to be 14–18 kb, and the lengths of the six mitochondrial genomes in this study were within this range [44]. In Alethinophidia, the mitochondrial genes of snakes hold two control regions (CR1, CR2), and their sequences are highly similar. In the species studied in this study, the length range of CR1 was 1019–1065 bp, and the length range of CR2 was 1011–1058 bp. The differences in the size of mtDNA are mainly caused by changes in the length of this region because that control region is the most susceptible to variation in length and sequence of vertebrate mtDNA.

The study showed that they exhibited a distinct high AT bias, which was highly similar to those of other Colubridae species [45,46,47]. AT preference is related to the geographical location and environmental temperature where the species lives [48]. In addition, the unbalanced use of the four bases can also lead to high A + T content [49]. The positive AT skew and negative GC skew was in alignment with the traits of most snake genomes and met the criteria for asymmetry in the mitochondrial gene composition of amniotes [50].

Among the 13 PCGs of all mitogenomes, only one gene (nad4) was located on the heavy strand, indicating that it had relative stability. Research has found that the vast majority genes use ATG as the initiator codon, which is regarded as the most common initiator codon in vertebrates [44]. Furthermore, the bulk of genes have incomplete stop codons, which has been repeatedly discovered in other animal mitogenomes [51,52,53]. Those incomplete stop codons can be converted into complete codons through post-transcriptional polyadenylation [54]. Codon usage patterns refer to the non-random selection phenomenon of synonymous codons (different codons encoding the same amino acid) during the translation process in organisms, where RSCU serves as a key metric to quantify this pattern by measuring the relative usage frequency of a specific codon within its synonymous codon family; moreover, codon usage pattern can judge the genetic relationship, and the closer the genetic relationship to a species, the more similar their RSCU values [55]. In this study, the RSCU values displayed a high degree of resemblance in codon preference among the six species, further proving their close genetic relationship.

The Ka/Ks analysis revealed that all 13 PCGs are under purifying selection (Ka/Ks < 1), consistent with their essential roles in mitochondrial function [56,57]. The Ka/Ks and Pi values of atp6 and atp8 were higher than those of other genes, indicating that mitochondrial ATPase subunits (atp6/atp8) often evolve rapidly due to their peripheral position in the electron transport chain complexes, which may tolerate more structural variations. cox1 and cox3 were the most conserved genes, likely because their catalytic roles in cytochrome c oxidase impose strict structural requirements. These patterns align with observations in Python snakes [58]. On the other hand, the high Ka/Ks ratio and Pi value of atp8 and atp6 also have been reported in other vertebrates [59].

Since mitochondrial tRNA genes are approximately the same size, they have a higher rate of evolution than nuclear tRNA genes do [60]. All genes except trnS1 and/or trnC could form a typical cloverleaf structure (Figure S3–S8). Since trnS1 lacked the dihydroxyuracil arm (D arm) and the trnC gene lacked the T-arm, they could not form a cloverleaf structure. The above phenomenon has also been found in other Colubridae species [61]. What is more, the lack of a D-arm or a T-arm in tRNA genes results in decreased peptide production, aminoacylation levels, and EF-Tu binding ability [62]. It is worth noting that a pseudogene trnP with a length of 53 bp was found between the mitochondrial gene trnl and the control region of *L. futsingensis*. Pseudogenes are non-functional or degenerated gene sequences, typically formed as inactive copies during evolution. The functional trnP gene is located next to CR1 (location in typical vertebrate mtDNA), while a pseudogene (trnP) may be present or absent near CR2. This phenomenon also exists in some snakes [63,64,65]. The other five species, considered primitive snakes, did not have the pseudogene trnP [66]. Different from the typical arrangement of tRNA genes in vertebrates, trnL in these six species had been moved from its original position between rrnL and nad1 to between CR2 and trnQ, which is common in Alethinophidia [64,67,68].

We further generated BI and ML trees with concatenated alignment of PCGs. In addition to the six newly sequenced species in this study, the other six *Lycodon* species in the phylogenetic tree were all species recorded on NCBI that have uploaded the complete mitochondrial genome sequence. Differently from previous studies, we used snakes’ mitochondrial whole-genome tree construction, while previous studies were mostly based on single-gene or multi-gene tree construction. In contrast, mitochondrial whole-gene tree construction has more biological evolution information and is more convincing than single-gene tree construction. The result indicated that the 12 species of genus *Lycodon* clustered in a monophyletic clade (Figure 4), which is consistent with the findings from previous phylogenetic studies [3]. *L. aulicus*, *L. gongshan*, *L. faciatus*, and *L. rustrati* were a monophyletic group, and *L. aulicus* and *L. faciatus* had the closest genetic relationship. According to other research results, *L. gongshan*, *L. faciatus*, and *L. rustrati* are still in the same monophyletic group, but *L. aulicus* is not in the same monophyletic group with them [3,14,69]. Similarly, the evolutionary positions of *L. aulicus* in previous studies were also different. This difference may be due to the different datasets used to construct the phylogenetic tree: previous studies built trees based on partial genomes, while ours were based on whole genomes. Consistently with other studies, *L. subcinctus*, *L. futstingensis*, *L. rosozonatus*, and the other four species were all on the same clade, and *L. subcinctus* was the sister of their five clade lines [3,14,69]. However, something different was that we found that *L. futstingensis* was the sister of the clade of five other species (*L. semicarinatus*, *L. flavozonatus*, *L. rosozonatus*, and *L. rufozonatus*), but other studies have shown that it and *L. semicarinatus* or *L. flavozonatus* were sisters of each other [3,70]. In summary, these differences remind us of the need to work hard to obtain more molecular data to determine phylogenetic relationships. There are 82 species in the genus *Lycodon*, but six species have been studied so far, and we have contributed to adding six species. However, it is far from the 82 species, so research on other undiscovered species is necessary in the future.

## 5. Conclusions

The mitogenomes of six *Lycodon* species (*L. subcinctus*, *L. rosozonatus*, *L. fasciatus*, *L. gongshan*, *L. futsingensis*, and *L. aulicus*) were newly sequenced and analyzed in this study. Structure and evolutionary analyses of the mitogenomes of *Lycodon* were conducted. The results showed that these six mitogenomes of the genus had similar structural characteristics and were biased toward A/T. In addition, the analysis showed that atp8 and atp6 evolved quickly, while cox1 and cox3 were relatively conserved genes, and the pseudogene trnP was found in *L. futsingensis*. Phylogenetic analysis indicated that the six species clustered together, but there are some differences in their genetic relationships compared with those from other studies. Therefore, it is necessary to improve the complete mitochondrial genome sample of the genus *Lycodon* and reconstruct a more comprehensive phylogenetic tree.

## Figures and Tables

**Table 1:** Sample information.

Species	Place of Collection
*L. subcinctus*	Zhexiang Town, Wangmo County, Guizhou Province, China
*L. rosozonatus*	Diaoluoshan National Forest Park, Lingshui Li Autonomous County, Hainan Province, China
*L. fasciatus*	Daweshan, Pingbian County, Yunnan Province, China
*L. gongshan*	Wulaoshan, Lincang City, Yunnan Province, China
*L. futsingensis*	Danzhu Township, Xianju County, Zhejiang Province, China
*L. aulicus*	Daweshan, Pingbian County, Yunnan Province, China

**Table 2:** Detailed information about gene content and composition of the six newly determined *Lycodon* mitochondrial genomes. The statistical results of the newly determined mitogenomes of *L. subcinctus*, *L. rosozonatus*, *L. fasciatus*, *L. gongshan*, *L. futsingensis*, and *L. aulicus*.

Gene	Position		Size (bp)	Start Codon	Stop Codon	Strand
	From	To				
trnF	1/1/1/1/1/1	65/66/67/64/67/67	65/66/67/64/67/67			H/H/H/H/H/H
rrn12	66/65/66/63/65/66	991/995/991/990/992/991	926/931/926/928/928/926			H/H/H/H/H/H
trnV	992/996/992/991/993/992	1055/1059/1055/1054/1056/1055	64/64/64/64/64/64			H/H/H/H/H/H
rrn16	1056/1060/1056/1060/1065/1056	2525/2533/2522/2516/2530/2522	1470/1474/1467/1467/1466/1467			H/H/H/H/H/H
nad1	2526/2534/2523/2517/2531/2523	3490/3497/3486/3481/3494/3486	965/964/964/965/964/964	ATA/ATA/ATA/ATA/ATA/ATA	TA-/T--/T--/TA-/T--/T--	H/H/H/H/H/H
trnl	3491/3498/3487/3482/3495/3487	3555/3563/3552/3547/3559/3552	65/66/66/66/65/66			H/H/H/H/H/H
trnL	4641/4575/4645/4601/4719/4645	4713/4647/4717/4673/4791/4717	73/73/73/73/73/73			H/H/H/H/H/H
trnQ	4715/4649/4719/4675/4793/4719	4785/4719/4789/4745/4863/4789	71/71/71/71/71/71			L/L/L/L/L/L
trnM	4787/4721/4791/4747/4865/4791	4850/4783/4854/4809/4927/4854	64/63/64/63/63/64			H/H/H/H/H/H
nad2	4851/4784/4855/4810/4928/4855	5877/5813/5884/5839/5957/5884	1027/1030/1030/1030/1030/1030	ATT/ATT/ATT/ATT/ATT/ATT	T--/T--/T--/T--/T--/T--	H/H/H/H/H/H
trnW	5878/5814/5885/5840/5858/5885	5942/5878/5948/5903/6022/5948	65/65/64/64/65/64			H/H/H/H/H/H
trnA	5943/5880/5951/5909/6024/5951	6007/5944/6015/5973/6088/6015	65/65/65/65/65/65			L/L/L/L/L/L
trnN	6008/5945/6016/5974/6089/6016	6079/6016/6087/6045/6160/6087	72/72/72/72/72/72			L/L/L/L/L/L
trnC	6115/6052/6122/6081/6195/6112	6174/6111/6181/6140/6254/6184	60/60/60/60/60/60			L/L/L/L/L/L
trnY	6175/6112/6182/6141/6255/6182	6236/6173/6244/6202/6316/6244	62/62/63/62/62/63			L/L/L/L/L/L
cox1	6238/6175/6246/6204/6318/6246	7783/7721/7792/7750/7864/7792	1546/1547/1547/1547/1547/1547	GTG/GTG/GTG/GTG/GTG/GTG	T--/T--/TA-/TA-/TA-/TA-	H/H/H/H/H/H
trnS2	7830/7767/7838/7796/7910/7838	7895/7832/7903/7861/7975/7903	66/66/66/66/66/66			L/L/L/L/L/L
trnD	7896/7833/7904/7862/7976/7904	7958/7895/7967/7924/8038/7967	63/63/64/63/63/64			H/H/H/H/H/H
cox2	7959/7896/7968/7925/8039/7968	8643/8580/8652/8609/8723/8652	685/685/685/685/685/685	ATG/ATG/ATG/ATG/ATG/ATG	T--/T--/T--/T--/T--/T--	H/H/H/H/H/H
trnK	8644/8581/8653/8610/8724/8653	8705/8643/8714/8671/8786/8714	62/63/63/62/63/62			H/H/H/H/H/H
atp8	8706/8645/8716/8673/8788/8716	8864/8803/8874/8831/8946/8874	159/159/159/159/159/159	ATG/ATG/ATG/ATG/ATG/ATG	TAA/TAA/TAA/TAA/TAA/TAA	H/H/H/H/H/H
atp6	8855/8794/8865/8822/8937/8865	9535/9474/9545/9502/9617/9545	681/681/681/681/681/681	ATG/ATG/ATG/ATG/ATG/ATG	TAA/TAA/TAA/TAA/TAA/TAA	H/H/H/H/H/H
cox3	9535/9474/9545/9502/9617/9545	10318/10257/10328/10285/10400/10328	784/784/784/784/784/784	ATG/ATG/ATG/ATG/ATG/ATG	T--/T--/T--/T--/T--/T--	H/H/H/H/H/H
trnG	10319/10258/10329/10286/10401/10329	10379/10318/10389/10346/10461/10389	61/61/61/61/61/61			H/H/H/H/H/H
nad3	10380/10319/10390/10347/10462/10390	10722/10661/10732/10689/10804/10732	343/343/343/343/343/343	ATT/ATT/ATT/ATT/ATT/ATT	T--/T--/T--/T--/T--/T--	H/H/H/H/H/H
trnR	10723/10662/10733/10690/10805/10733	10786/10726/10797/10754/10869/10797	64/65/65/65/65/65			H/H/H/H/H/H
nad4L	10787/10727/10798/10755/10870/10798	11077/11017/11088/11045/11160/11088	291/291/291/291/291/291	ATG/ATG/ATG/ATG/ATG/ATG	TAA/TAA/TAA/TAA/TAA/TAA	H/H/H/H/H/H
nad4	11077/11017/11088/11045/11160/11088	12413/12354/12424/12381/12497/12424	1337/1338/1337/1337/1338/1337	ATG/ATG/ATG/ATG/ATG/ATG	TA-/TAA/TA-/TA-/TAA/TA-	H/H/H/H/H/H
trnH	12414/12355/12425/12382/12498/12425	12479/12420/12488/12446/12563/12488	66/66/64/65/66/64			H/H/H/H/H/H
trnS1	12480/12421/12489/12447/12564/12489	12536/12478/12545/12503/12621/12545	57/58/57/57/58/57			H/H/H/H/H/H
trnL	12534/12476/12543/12501/12619/12543	12604/12546/12613/12571/12689/12613	71/71/71/71/71/71			H/H/H/H/H/H
nad5	12605/12547/12614/12572/12690/12614	14374/14319/14383/14341/14462/14383	1770/1773/1770/1770/1773/1770	ATG/ATG/ATG/ATG/ATG/ATG	TAA/TAA/TAA/TAA/AGG/TAA	H/H/H/H/H/H
nad6	14370/14311/14379/14337/14454/14379	14873/14808/14882/14837/14954/14882	504/498/504/501/501/504	ATG/ATG/ATG/ATG/ATG/ATG	AGG/AGG/AGG/AGG/AGA/AGG	L/L/L/L/L/L
trnE	14883/14818/14892/14847/14964/14892	14944/14879/14953/14908/15025/14953	62/62/62/62/62/62			L/L/L/L/L/L
Cyt b	14945/14880/14954/14909/15028/14954	16061/15996/16070/16025/16144/16070	1117/1117/1117/1117/1117/1117	ATG/ATG/ATG/ATG/ATG/ATG	T--/T--/T--/T--/T--/T--	H/H/H/H/H/H
trnT	16062/15997/16071/16026/16145/16071	16125/16062/16135/16089/16210/16135	64/66/65/64/66/65			H/H/H/H/H/H
trnP	16126/16063/16136/16090/16211/16136	16188/16124/16196/16151/16272/16197	63/62/61/62/62/62			L/L/L/L/L/L
CR1	16189/16125/16197/16152/16273/16198	17235/17143/17261/17175/17298/17262	1047/1019/1065/1024/1026/1065			
CR2	3555/3564/3553/3548/3560/3553	4640/4574/4644/4600/4718/4644	1086/1011/1092/1053/1158/1092			

**Table 3:** The location, size, and number of intergenic spacers of the six newly sequenced mitogenomes.

Species	trnL2-trnQ (bp)	trnQ-trnM (bp)	trnW-trnA (bp)	trnN-trnC (bp)	trnY-cox1 (bp)	cox1-trnS2 (bp)	trnK-atp8 (bp)	nad6-trnE (bp)	trnE-cytb (bp)	Count
*L. subcinctus*	1	1	/	35	1	46	/	9	/	6
*L. rosozonatus*	1	1	1	35	1	45	1	9	/	8
*L fasciatus*	1	1	5	35	1	45	1	9	/	8
*L. gongshan*	1	1	2	34	1	45	1	9	/	8
*L. futsingensis*	1	1	1	34	1	45	1	9	2	9

**Table 4:** The location, size, and number of overlap regions of the six newly sequenced mitogenomes.

Species	atp8-atp6 (bp)	atp6-cox3 (bp)	trnS1-trnL1 (bp)	nad5-nad6 (bp)	nad4L-nad4 (bp)	trnF-rrnS (bp)	Count
*L. subcinctus*	10	1	3	5	1	0	5
*L. rosozonatus*	10	1	3	9	1	2	6
*L. fasciatus*	10	1	3	5	1	2	6
*L. gongshan*	10	1	3	5	1	2	6
*L. futsingensis*	10	1	3	9	1	3	6
*L. aulicus*	10	1	3	5	1	2	6

**Table 5:** Base compositions of the complete genomes, PCGs, rRNAs, and tRNAs of the six newly sequenced mitogenomes.

Species	Whole Genome		AT Skew	GC Skew	PCGs		AT Skew	GC Skew	tRNAs		AT Skew	GC Skew	rRNAs		AT Skew	GC Skew
	Size (bp)	AT (%)			Size (bp)	AT (%)			Size (bp)	AT (%)			Size (bp)	AT (%)		
*L. subcinctus*	17,235	58.6	0.173	−0.387	10,695	58.2	0.176	−0.436	904	58.1	0.185	−0.150	2396	58.1	0.340	−0.227
*L. rosozonatus*	17,143	59.4	0.165	−0.380	10,704	59.3	0.160	−0.426	910	58.1	0.174	−0.168	2405	59.0	0.321	−0.208
*L. fasciatus*	17,261	58.5	0.150	−0.374	10,698	58.3	0.146	−0.416	907	58.1	0.184	−0.163	2393	58.6	0.323	−0.213
*L. gongshan*	17,175	58.2	0.154	−0.370	10,698	57.7	0.149	−0.409	902	58.1	0.172	−0.138	2385	58.7	0.317	−0.216
*L. futsingensis*	17,298	59.0	0.159	−0.384	10,704	59.4	0.148	−0.424	910	57.8	0.171	−0.167	2394	59.2	0.315	−0.209
*L. aulicus*	17,262	58.5	0.150	−0.374	10,698	58.3	0.146	−0.414	907	58.1	0.184	−0.163	2393	58.6	0.323	−0.213

**Table 6:** Codon number and RSCU in protein-coding genes (PCGs) of *L. subcinctus*, *L. rosozonatus*, *L. fasciatus*, *L. gongshan*, *L. futsingensis*, and *L. aulicus*.

AA	Codon	Count	RSCU	AA	Codon	Count	RSCU
Phe	UUU(F)	85/89/84/96/86/87	0.86/0.88/0.83/0.92/0.87/0.88	Tyr	UAU(Y)	38/27/38/40/41/40	0.74/0.51/0.7/0.75/0.77/0.75
Phe	UUC(F)	113/114/119/112/111/110	1.14/1.12/1.17/1.08/1.13/1.12	Tyr	UAC(Y)	65/78/71/64/66/66	1.26/1.49/1.3/1.25/1.23/1.25
Leu	UUA(L)	86/81/84/81/80/80	0.90/0.87/0.90/0.88/0.83/0.83	Stop codon	UAA	4/4//4/4/4/4	3.20/2.67/3.20/2.67/3.2/3.20
Leu	UUG(L)	20/20/17/23/19/19	0.21/0.22/0.18/0.25/0.20/0.20	Stop codon	UAG	0/0/0/0/0/0	0/0/0/0/0/0
Leu	CUU(L)	59/57/69/70/76/78	0.62/0.61/0.74/0.76/0.79/0.81	His	CAU(H)	13/20/15/13/15/17	0.25/0.37/0.28/0.25/0.28/0.31
Leu	CUC(L)	63/70/64/55/70/69	0.66/0.75/0.68/0.59/0.73/0.72	His	CAC(H)	90/88/92/91/93/92	1.75/1.63/1.72/1.75/1.72/1.69
Leu	CUA(L)	316/297/283/294/286/286	3.32/3.20/3.02/3.18/2.98/2.98	Gln	CAA(Q)	91/86/83/84/86/86	1.88/1.79/1.71/1.79/1.81/1.81
Leu	CUG(L)	27/32/46/32/45/44	0.28/0.34/0.49/0.35/0.47/0.46	Gln	CAG(Q)	6/10//14/10/9/9	0.12/0.21/0.29/0.21/0.19/0.19
Ile	AUU(I)	121/149/131/150/132/133	0.81/0.96/0.91/0.99/0.91/0.91	Asn	AAU(N)	34/46/32/44/33/33	0.45/0.58/0.44/0.55/0.44/0.44
Ile	AUC(I)	177/161/158/154/158/158	1.19/1.04/1.09/1.01/1.09/1.09	Asn	AAC(N)	117/112/115/117/116/116	1.55/1.42/1.56/1.45/1.56/1.56
Met	AUA(M)	232/241/220/242/228/229	1.74/1.75/1.66/1.72/1.70/1.71	Lys	AAA(K)	91/95/87/90/86/87	1.80/1.90/1.74/1.82/1.74/1.76
Met	AUG(M)	35/34/45/39/40/39	0.26/0.25/0.34/0.28/0.30/0.29	Lys	AAG(K)	10/5//13/9/13/12	0.20/0.10/0.26/0.18/0.26/0.24
Val	GUU(V)	30/33/51/40/55/55	0.75/0.78/1.15/0.98/1.30/1.30	Asp	GAU(D)	11/13/14/13/12/12	0.37/0.47/0.50/0.47/0.43/0.43
Val	GUC(V)	38/35/38/34/25/25	0.95/0.83/0.86/0.83/0.59/0.59	Asp	GAC(D)	49/42/42/42/44/44	1.63/1.53/0.50/1.53/1.57/1.57
Val	GUA(V)	70/74/68/67/70/70	1.75/1.75/1.54/1.63/1.66/1.66	Glu	GAA(E)	74/80/71/83/74/74	1.61/1.70/1.58/1.78/1.61/1.61
Val	GUG(V)	22/27/20/23/19/19	0.55/0.64/0.45/0.56/0.45/0.45	Glu	GAG(E)	18/14/19/10/18/18	0.39/0.30/0.42/0.22/0.39/0.39
Ser	UCU(S)	44/39/30/39/28/27	1.04/0.92/0.69/0.89/0.63/0.61	Cys	UGU(C)	12/13/14/12/14/14	0.74/0.73/0.88/0.73/0.85/0.85
Ser	UCC(S)	51/59/63/64/73/72	1.20/1.39/1.45/1.47/1.65/1.64	Cys	UGC(C)	22/21/18/21/19/19	1.26/1.27/1.12/1.27/1.15/1.15
Ser	UCA(S)	106/103/107/107/110/109	2.49/2.42/2.46/2.45/2.48/2.48	Trp	UGA(W)	77/87/80/83/81/80	1.60/1.76/1.63/1.69/1.65/1.63
Ser	UCG(S)	7/9//6/5/5/6	0.16/0.21/0.14/0.11/0.11/0.14	Trp	UGG(W)	12/19/18/15/17/18	0.40/0.24/0.37/0.31/0.35/0.37
Pro	CCU(P)	14/20/16/18/19/18	0.29/0.41/0.33/0.38/0.40/0.38	Arg	CGU(R)	4/3//5/8/5/5	0.27/0.21/0.33/0.53/0.33/0.33
Pro	CCC(P)	49/33/60/47/44/45	1.02/0.68/1.23/0.98/0.94/0.96	Arg	CGC(R)	12/13/14/9/13/13	0.80/0.90/0.93/0.60/0.85/0.85
Pro	CCA(P)	125/128/109/123/118/118	2.59/2.65/2.24/2.56/2.51/2.51	Arg	CGA(R)	37/37/33/38/37/37	2.47/2.55/2.20/2.53/2.43/2.43
Pro	CCG(P)	5/12//10/4/7/7	0.10/0.25/0.21/0.08/0.15/0.15	Arg	CGG(R)	7/5//8/5/6/6	0.47/0.34/0.53/0.33/0.39/0.39
Thr	ACU(T)	40/51/50/56/48/47	0.35/0.47/0.45/0.52/0.44/0.44	Ser	AGU(S)	11/12//12/13/9/9	0.26/0.28/0.28/0.30/0.20/0.20
Thr	ACC(T)	186/168/176/157/160/158	1.61/1.56/1.59/1.46/1.48/1.46	Ser	AGC(S)	36/33/43/34/41/41	0.85/0.78/0.99/0.78/0.92/0.93
Thr	ACA(T)	222/208/207/204/212/212	1.92/1.93/1.87/1.90/1.96/1.96	Stop codon	AGA	1/1/0/1/0/0	0.80/0.67/0/0.67/0/0
Thr	ACG(T)	5/15/9/12/13/15	0.13/0.05/0.08/0.11/0.12/0.14	Stop codon	AGG	0/1//1/1/1/1	0/0.67/0.80/0.67/0.80/0.80
Ala	GCU(A)	25/39/39/38/45/45	0.45/0.68/0.66/0.65/0.73/0.73	Gly	GGU(G)	22/29/14/23/22/22	0.44/0.59/0.28/0.47/0.44/0.44
Ala	GCC(A)	105/93/100/96/104/106	1.88/1.61/1.68/1.64/1.70/1.72	Gly	GGC(G)	58/42/52/55/56/56	1.15/0.85/1.05/1.12/1.13/1.13
Ala	GCA(A)	85/97/93/96/91/91	1.52/1.68/1.56/1.64/1.49/1.47	Gly	GGA(G)	76/87/67/74/75/75	1.51/1.77/1.35/1.51/1.51/1.51
Ala	GCG(A)	9/2//6/4/5/5	0.16/0.03/0.10/0.07/0.08/0.08	Gly	GGG(G)	45/39/66/44/46/46	0.90/0.79/1.33/0.90/0.92/0.92

## Data Availability

The mitogenome was deposited at NCBI with the accession number PRJNA1232943.

## Supplementary Materials

The following supporting information can be downloaded at: https://www.mdpi.com/article/10.3390/genes16050493/s1, Figure S1: The type and count of simple sequence repeats (SSRs) of *L. subcinctus*, *L. rosozonatus*, *L. fasciatus*, *L. gongshan*, *L. futsingensis*, and *L. aulicus*; Figure S2: The length and count of dispersed repeats of *L. subcinctus*, *L. rosozonatus*, *L. fasciatus*, *L. gongshan*, *L. futsingensis*, and *L. aulicus*.; Figure S3: Putative secondary structures of tRNAs from the *L. subcinctus;* Figure S4: Putative secondary structures of tRNAs from the *L. rosozonatus*; Figure S5: Putative secondary structures of tRNAs from the *L. fasciatus*; Figure S6: Putative secondary structures of tRNAs from the *L. gongshan*; Figure S7: Putative secondary structures of tRNAs from the *L. futsingensis*; Figure S8: Putative secondary structures of tRNAs from the *L. aulicus*.
