# Supplementary material for: Mitochondrial Genomes of Six Snakes (Lycodon) and Implications for Their Phylogeny
Source: Genes (Basel). 2025 Apr 26;16(5):493. doi: 10.3390/genes16050493 (PMC12110799; doi:10.3390/genes16050493)
Supplement: Supplementary file 1 [file genes-16-00493-s001.zip › genes-3566532-supplementary.pdf]

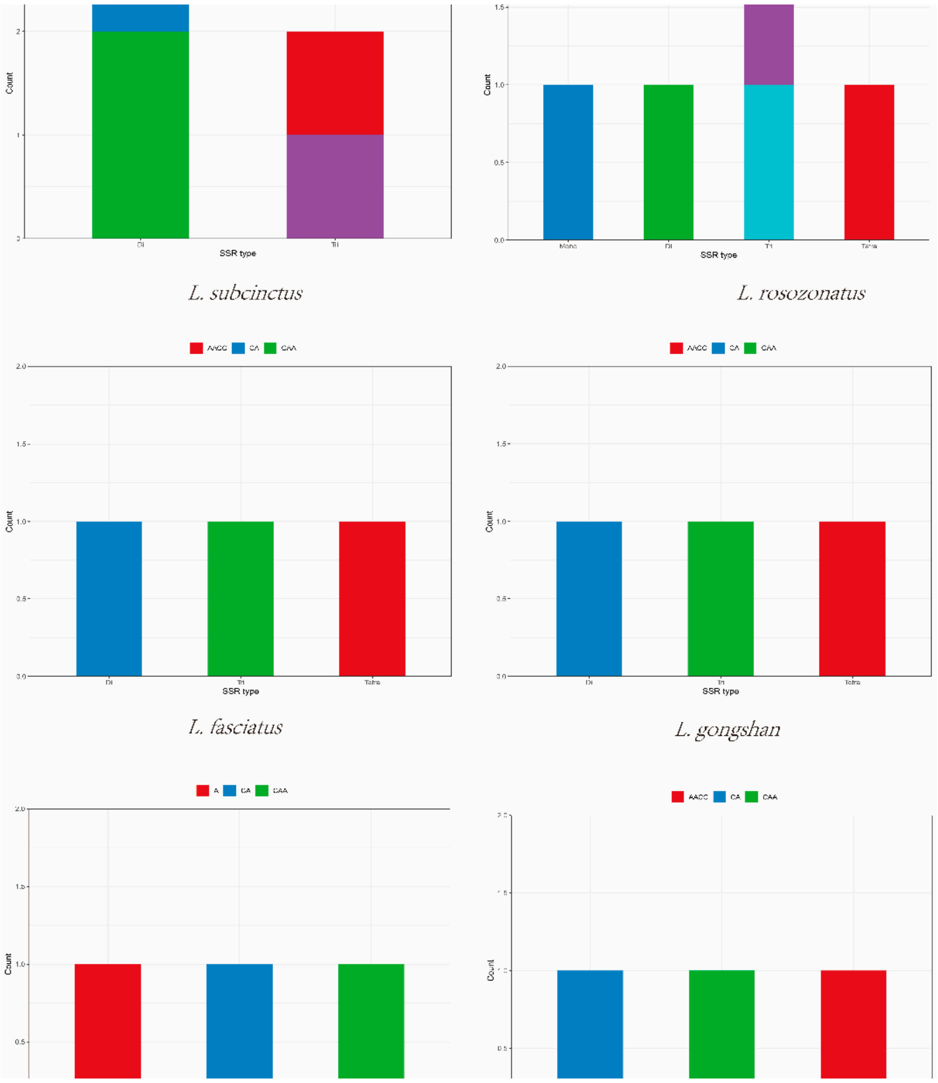

Figure S1. The type and count of simple sequence repeats (SSRs) of *L. subcinctus, L. rosozonatus, L. fasciatus, L. gongshan, L. futsingensis, and L. aulicus.*

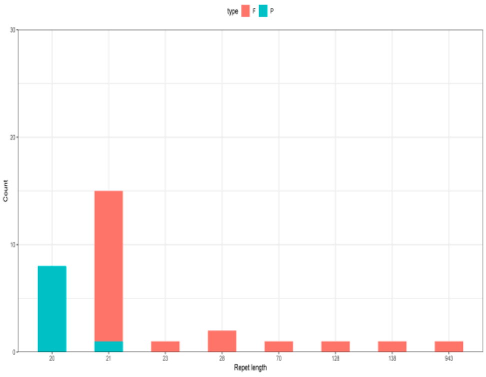

L. subcinctus

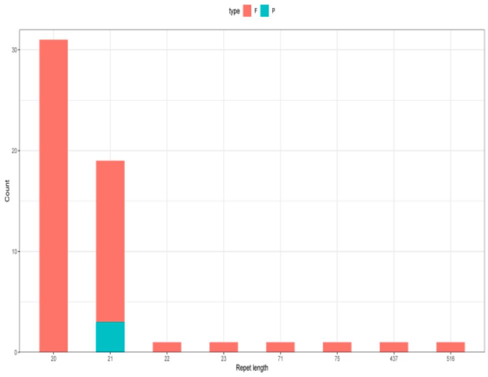

L. rosozonatus

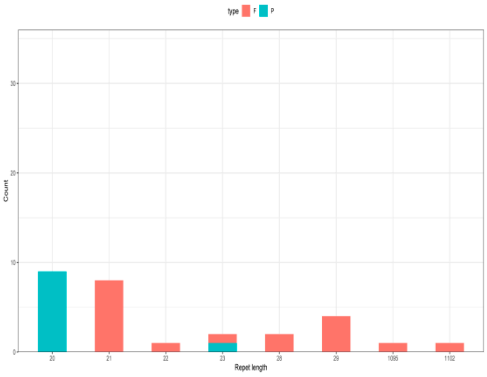

L. fasciatus

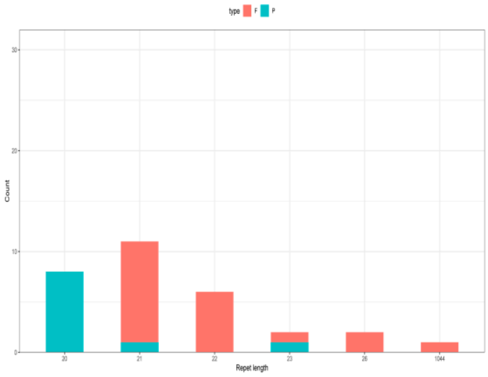

L. gongshan

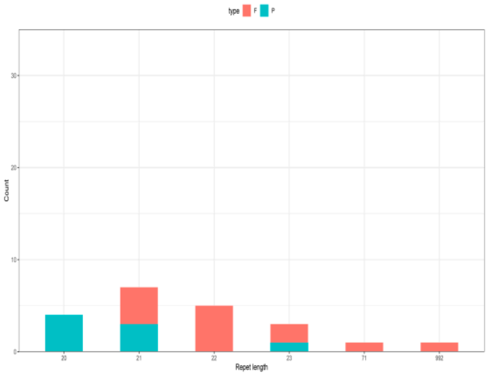

L. futsingensis

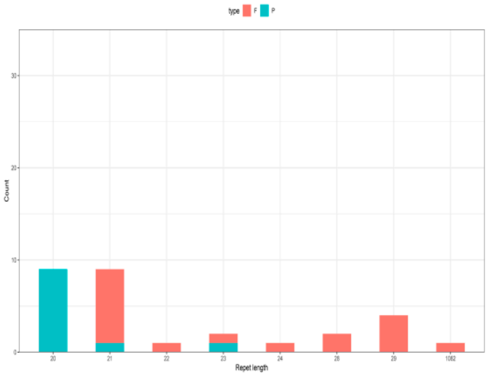

L. aulicus

Figure S2. The length and count of dispersed repeats of *L. subcinctus, L. rosozonatus, L. fasciatus, L. gongshan, L. futsingensis, and L. aulicus.*

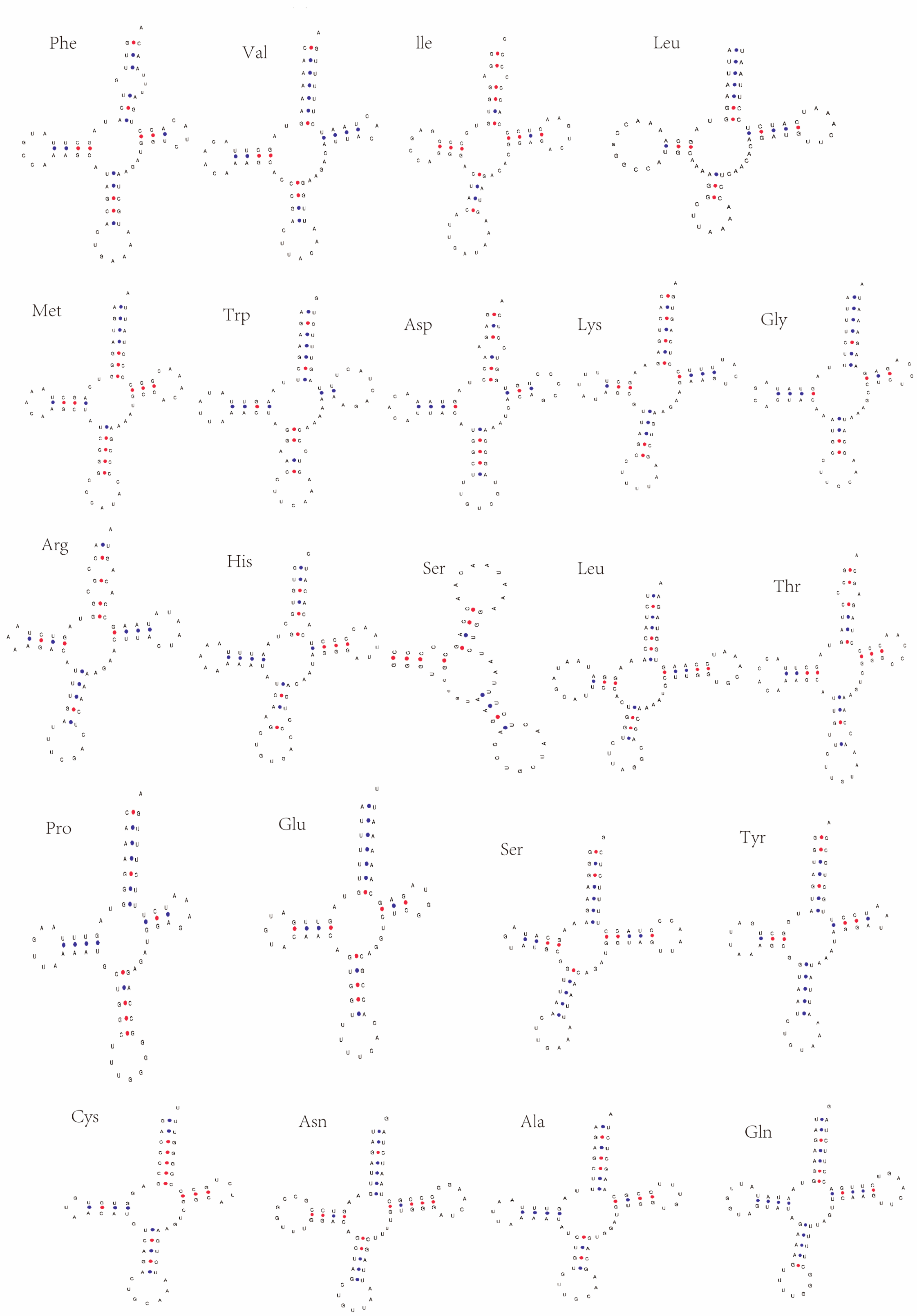

Figure S3. Putative secondary structures of tRNAs from the L. subcinctus.

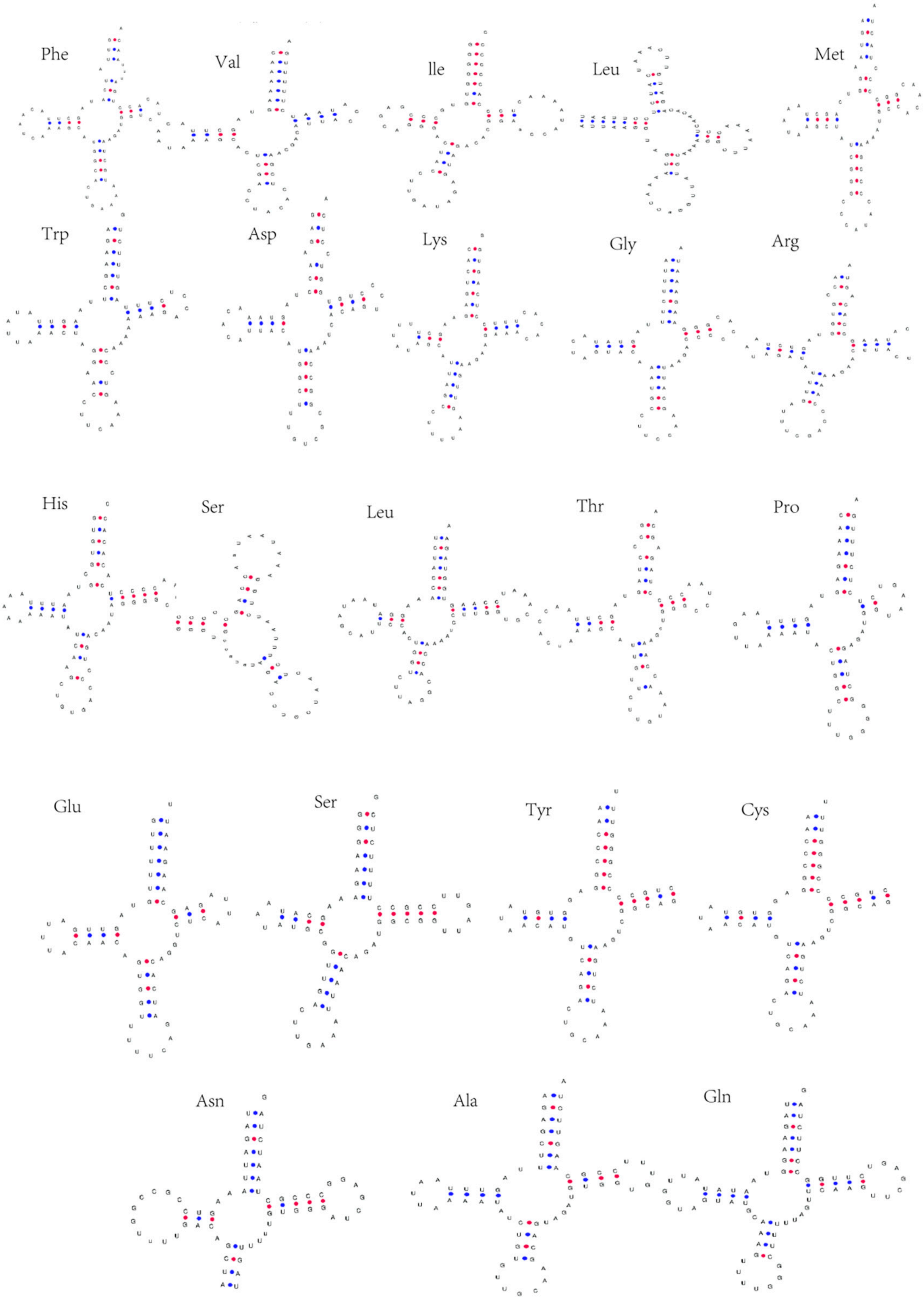

Figure S4. Putative secondary structures of tRNAs from the *L. rosozonatus*

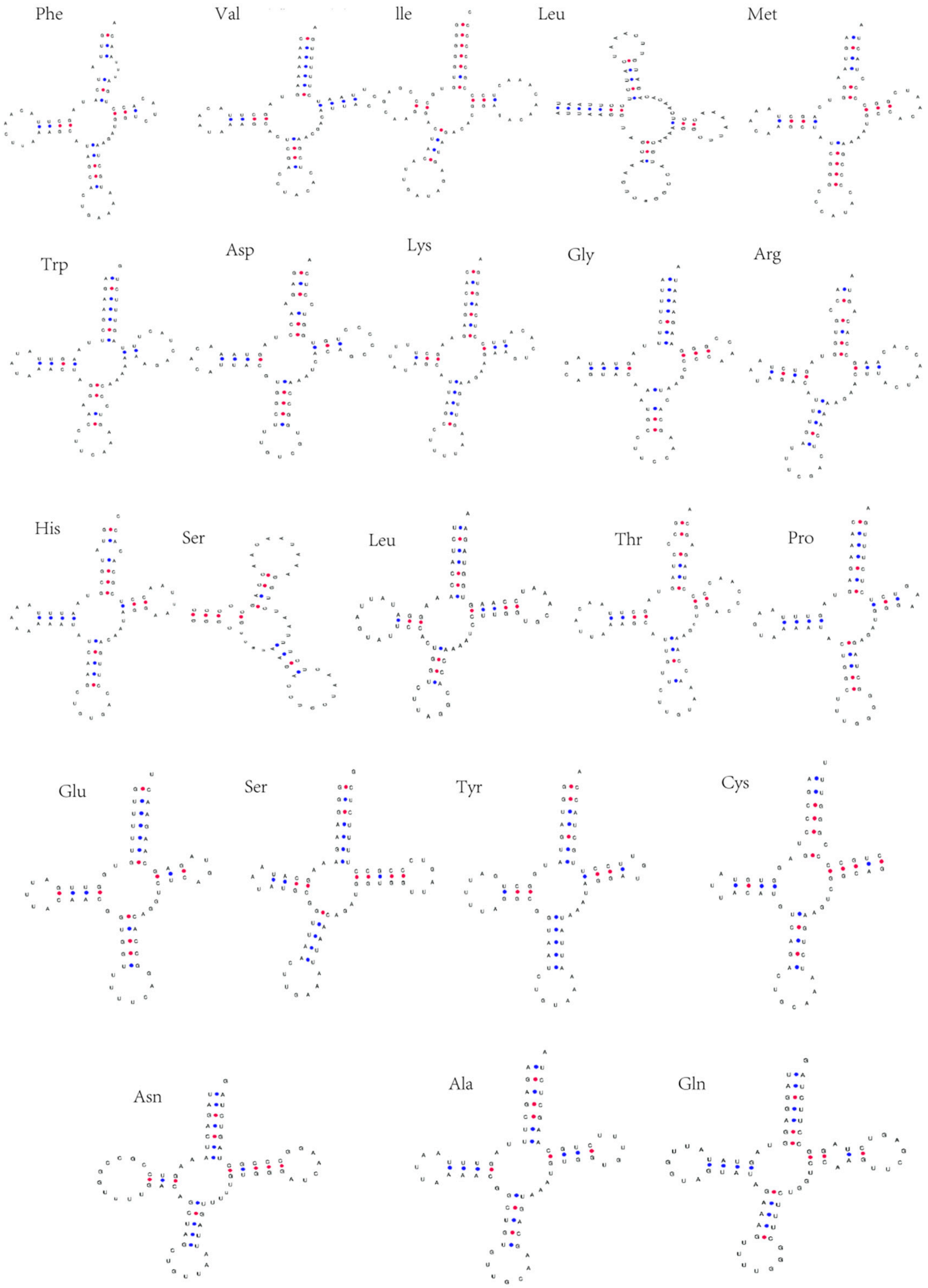

Figure S5. Putative secondary structures of tRNAs from the *L. fasciatus***.**

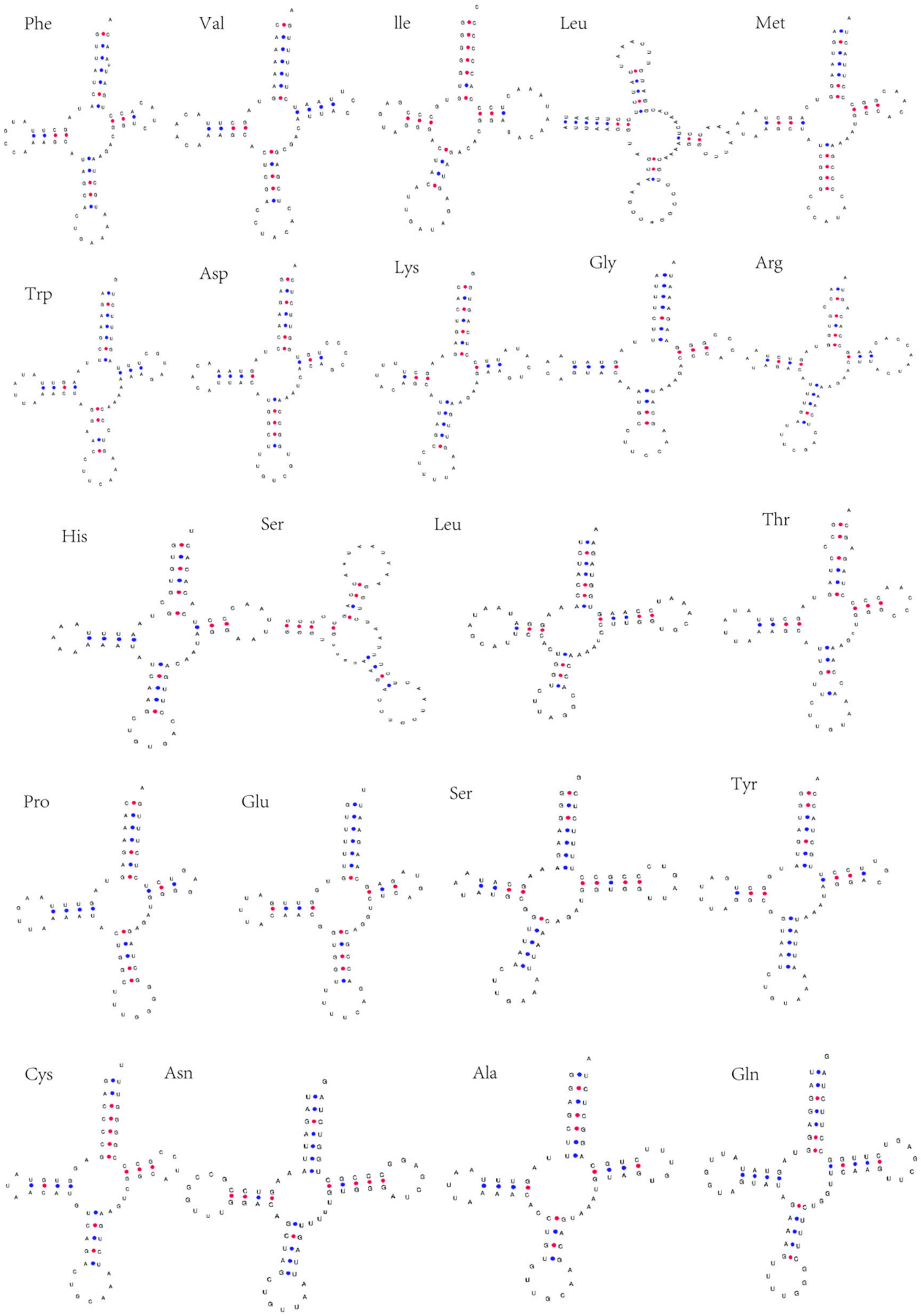

Figure S6. Putative secondary structures of tRNAs from the *L. gongshan*.

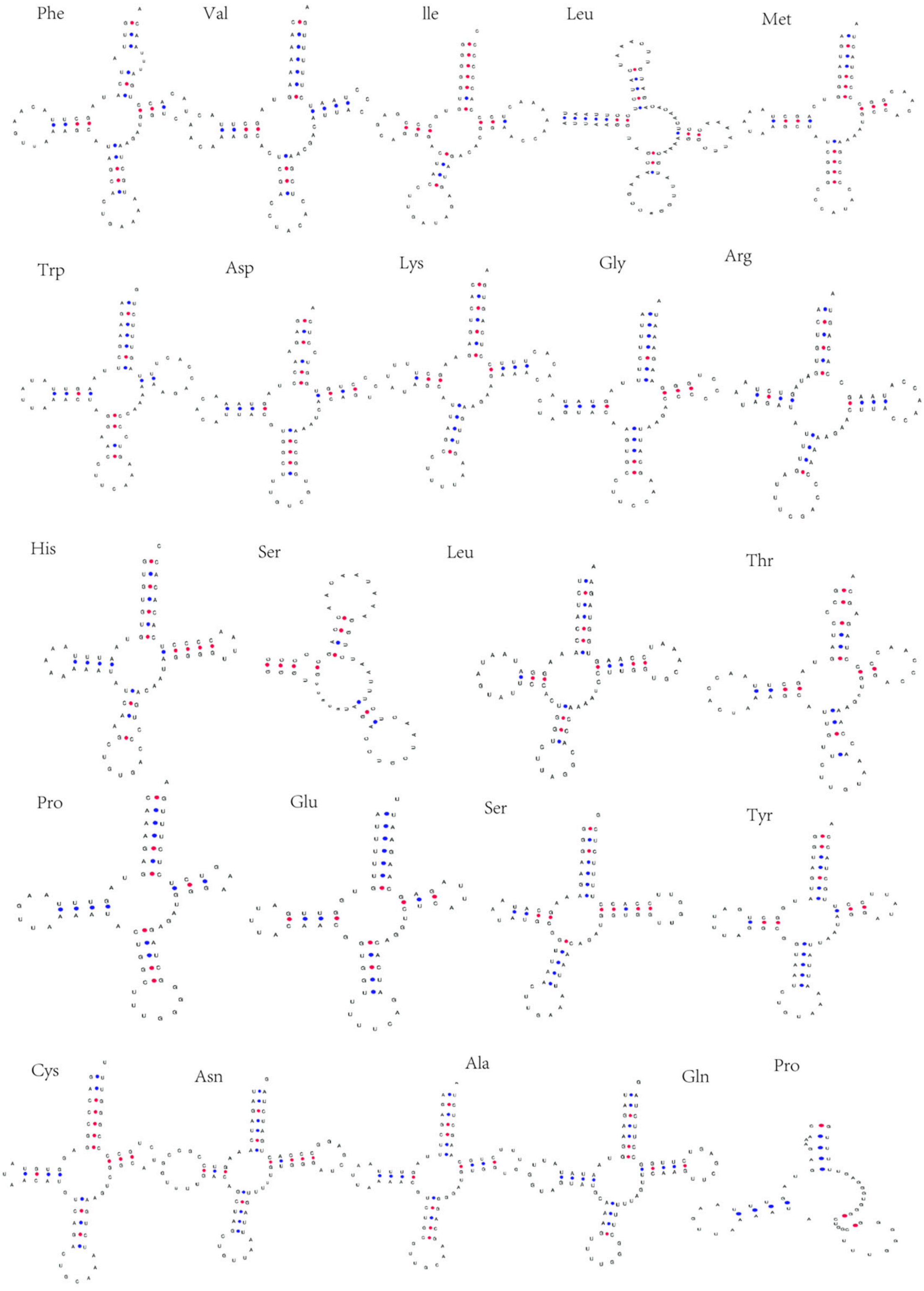

Figure S7. Putative secondary structures of tRNAs from the *L. futsingensis.*

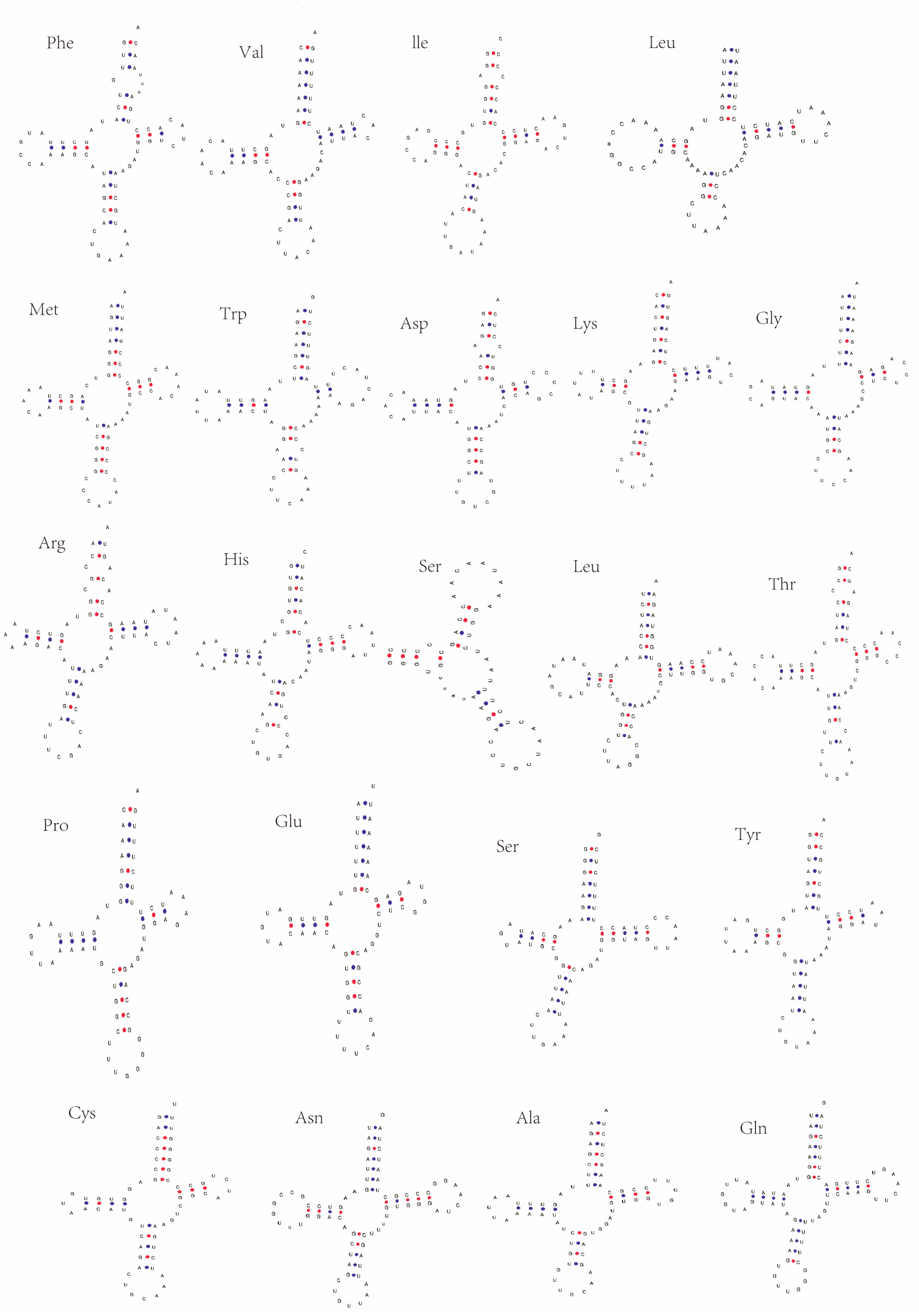

Figure S8. Putative secondary structures of tRNAs from the *L. aulicus*.
